# Hybrids of amphibian chytrid show high virulence in native hosts

**DOI:** 10.1038/s41598-018-27828-w

**Published:** 2018-06-25

**Authors:** S. E. Greenspan, C. Lambertini, T. Carvalho, T. Y. James, L. F. Toledo, C. F. B. Haddad, C. G. Becker

**Affiliations:** 1 Department of Biological Sciences, The University of Alabama, Tuscaloosa, AL 35487 USA; 2 Laboratório de História Natural de Anfíbios Brasileiros (LaHNAB), Departamento de Biologia Animal, Universidade Estadual de Campinas, Campinas, SP 13083-862 Brazil; 3 Department of Ecology and Evolutionary Biology, University of Michigan, Ann Arbor, MI 48109 USA; 4 Department of Zoology and Aquaculture Center (CAUNESP), Universidade Estadual Paulista, Rio Claro, SP 13506-900 Brazil

## Abstract

Hybridization of parasites can generate new genotypes with high virulence. The fungal amphibian parasite *Batrachochytrium dendrobatidis* (*Bd*) hybridizes in Brazil’s Atlantic Forest, a biodiversity hotspot where amphibian declines have been linked to *Bd*, but the virulence of hybrid genotypes in native hosts has never been tested. We compared the virulence (measured as host mortality and infection burden) of hybrid *Bd* genotypes to the parental lineages, the putatively hypovirulent lineage *Bd*-Brazil and the hypervirulent Global Pandemic Lineage (*Bd*-GPL), in a panel of native Brazilian hosts. In *Brachycephalus ephippium*, the hybrid exceeded the virulence (host mortality) of both parents, suggesting that novelty arising from hybridization of *Bd* is a conservation concern. In *Ischnocnema parva*, host mortality in the hybrid treatment was intermediate between the parent treatments, suggesting that this species is more vulnerable to the aggressive phenotypes associated with *Bd*-GPL. *Dendropsophus minutus* showed low overall mortality, but infection burdens were higher in frogs treated with hybrid and *Bd*-GPL genotypes than with *Bd*-Brazil genotypes. Our experiment suggests that *Bd* hybrids have the potential to increase disease risk in native hosts. Continued surveillance is needed to track potential spread of hybrid genotypes and detect future genomic shifts in this dynamic disease system.

## Introduction

The host-parasite dynamic is a classic example of an evolutionary arms race; hosts face pressure to evolve defenses against parasites, while parasites face pressure to overcome host defenses^1,2^. Compared to vertebrate hosts, parasites have the advantages of short generation times and large population sizes, which may promote rapid rates of evolution^3^. In many cases, vertebrate hosts overcome parasite pressure through sexual recombination, which allows for the forming of new and potentially beneficial gene combinations^4–6^. However, many parasites also possess dynamic genomes, further increasing their evolutionary potential^7,8^. Pathogen recombination through hybridization occurs when previously isolated lineages come into contact, a process which has intensified in our increasingly globalized world^7,9–11^. As a result, hybridization of parasites is an emerging concern for biodiversity conservation^9,12,13^.

Traits of hybrid parasites may differ in various ways from the parental lineages^10,14^. One possible outcome of hybridization is ‘hybrid vigor’ in which phenotypes exceed the ranges of the parent populations through mechanisms such as additive, complementary, or dominance gene interactions^14–17^. Disease progression can change rapidly to the detriment of hosts if fitness components such as parasite fecundity, infectivity, transmission, virulence, reproductive rate, ability to exploit novel host resources, or ability to evade host immunity are enhanced through hybridization^13,18^. For example, the mobility of a hybrid cucumber mosaic virus (genus *Cucumovirus*) was enhanced by a protein from the congeneric tomato aspermy virus^19^ and the viability and replicability of a hybrid avian influenza virus were enhanced by a protein segment from a human influenza virus^20^. Similarly, hybrids of the fungal insect pathogen *Beauveria* expressed the unique insecticidal functions of both parents, increasing the biocontrol efficiency against moth and beetle crop pests^21,22^. In addition, enhanced physiological traits such as tolerance of heat or host toxicity could allow for expansion of the geographic ranges or host specificities of parasites^16^. For instance, in the fungal plant pathogen *Phytophthora*, hybrids colonized an expanded array of hosts compared to the parent fungi^10,23^.

Alternatively, hybridization may produce phenotypes that are intermediate between the parental populations, a form of ‘hybrid breakdown’ that results from incompatibilities between interacting genes^17^. For instance, the proportion of deaths of guinea pigs infected with *E*. *coli*-*Shigella* hybrids was intermediate between those of guinea pigs infected with the parental bacteria^24^. Similarly, the average time until death in mice infected with a hybrid pseudorabies virus (genus *Varicellovirus*) was intermediate between those of mice infected with the parental viruses^25^. First generation crosses often show hybrid vigor, whereas later generation crosses often show hybrid breakdown because of disruption of co-adapted gene complexes^26^.

The fungal parasite *Batrachochytrium dendrobatidis* (*Bd*) causes the potentially lethal amphibian disease chytridiomycosis^27^. During its complex evolutionary history, *Bd* diverged into multiple genetic lineages which primarily reproduce clonally^28^. Epizootic outbreaks of chytridiomycosis are predominantly associated with spread of the most recently derived lineage of *Bd*, termed the Global Panzootic Lineage (*Bd*-GPL). These outbreaks occurred in the Andes^29^, Brazil^30^, Central America^31^, the Caribbean^32^, California^33^, Spain^34^, Cameroon^35^, New Zealand^36^, and eastern Australia^37^, mostly in the 1970s–2000s. In contrast to *Bd*-GPL, *Bd* lineages of Brazilian^38^, Chinese^39^, Japanese^40^, Korean^41^, Swiss, and South African^32^ origin diverged early in the evolutionary history of *Bd* and have not been directly linked to host population declines. Early diverging *Bd* lineages show high genetic structure, indicating long-term presence of *Bd* in select geographic areas^28,39–42^. Moreover, low incidence, prevalence, host burden, and host mortality associated with some ancestral lineages^32,39–41,43,44^, despite environmental suitability for *Bd*^43,45^, has led to the hypothesis that older *Bd* lineages are locally hypovirulent toward native hosts as an outcome of long-term co-evolution^41–43,46^. However, controlled studies have not been undertaken to determine the relative virulence of global and putatively enzootic *Bd* genotypes across panels of local hosts.

The Atlantic Forest of southeastern Brazil is a major amphibian biodiversity hotspot^47^, despite high levels of deforestation and forest fragmentation^48,49^, and is the only known region in which divergent lineages of *Bd* coexist and hybridize^38,42^. The enzootic Brazilian lineage of *Bd* (*Bd*-Brazil) occurs in the southern Atlantic Forest, where it appears to have evolved with a relatively small group of endemic host species against a backdrop of specific microclimatic conditions^42^. In contrast, *Bd*-GPL appears to be effective at dispersing across the microclimatically diverse landscape of this region and infects a wide array of native and introduced amphibian hosts^42^, despite estimates that it was introduced to the Atlantic Forest only within the last few centuries^50^. Hybrid genotypes between *Bd*-Brazil and *Bd*-GPL were recently detected in a mountain range within the geographic range of *Bd*-Brazil^38,42^. Amphibian declines and extirpations in the Atlantic Forest have been linked to *Bd*^30^, but it is unknown whether outbreaks resulted from the introduction of *Bd*-GPL, an increase in virulence of *Bd*-Brazil, the emergence of GPL-Brazil hybrids, or some combination of these factors^30^. Thus, testing the relative virulence of these genotypes is a research priority, especially considering that the section of the Atlantic Forest in which amphibian declines and extirpations were most strongly linked to *Bd* overlaps with the hybrid zone^30^.

Our aim was to compare the virulence (ability to cause harm to the host) of hybrid *Bd* genotypes to the parental lineages *Bd*-Brazil and *Bd*-GPL, in native Brazilian hosts. In keeping with the hypothesis that *Bd*-Brazil shared a longer co-evolutionary history with Brazilian frog species, we predicted that *Bd*-Brazil would have lower virulence in native Brazilian frog species than *Bd*-GPL and hybrid genotypes. The relative virulence of *Bd*-GPL and hybrid genotypes were more difficult to predict because the influence of genetic recombination on *Bd* virulence factors has never been tested, so we proposed two possible hypotheses. First, hybrid genotypes could have intermediate virulence compared to the parental lineages. Second, hybrid genotypes could have higher virulence than both parental lineages. Under controlled laboratory conditions, we inoculated two relatively *Bd*-intolerant direct-developing frog species (*Brachycephalus ephippium* and *Ischnocnema parva*) and one more tolerant aquatic-breeding frog species (*Dendropsophus minutus*) with *Bd* isolates representing *Bd*-Brazil, *Bd*-GPL, and hybrids. We measured relative virulence by comparing mortality rates and infection burdens among treatments and considered the implications of our results for the evolutionary future of this ecologically important parasite and its diverse amphibian hosts.

## Results

### Brachycephalus ephippium *and* I. parva (*host species with direct development*)

**Figure 1:**
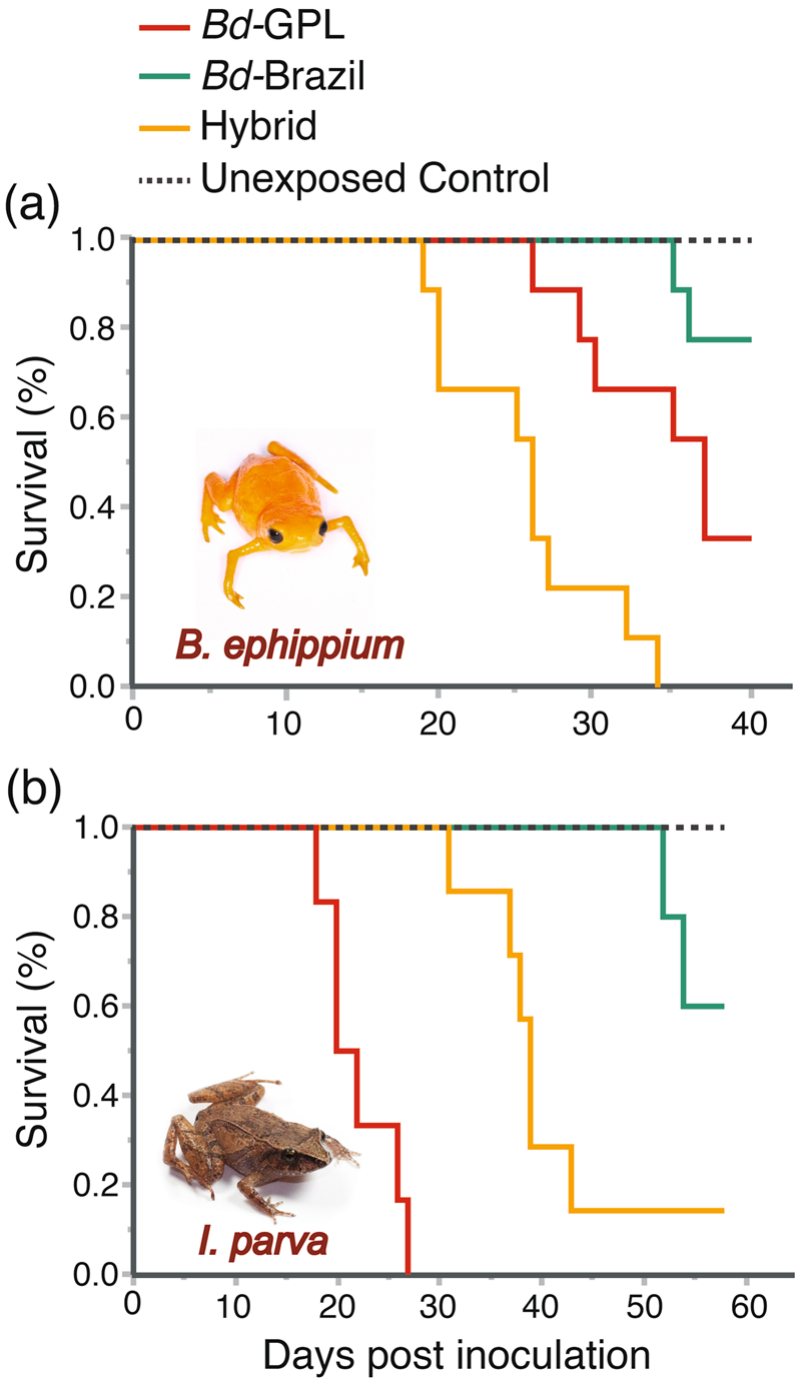
Survival curves for *Brachycephalus ephippium* (**a**) and *Ischnocnema parva* (**b**) exposed to *Bd*-GPL, *Bd*-Brazil and hybrid genotypes. Dashed line depicts unexposed control group.

The ranked mortality by treatment (from highest to lowest) for *B*. *ephippium* was: hybrid, *Bd*-GPL, and *Bd*-Brazil. (*χ*^2^ = 33.938; d.f. = 3; *p* < 0.0001; Fig. 1a). Mortality in *B*. *ephippium* exposed to *Bd*-Brazil was similar to the control (*p* = 0.100; Fig. 1a). Mortality was first observed in frogs exposed to *Bd*-Brazil on day 35 and 2/9 frogs (22%) died by the end of the experiment on day 40 (Fig. 1a). In contrast, frogs exposed to *Bd*-GPL (*p* = 0.002) and the hybrid (*p* < 0.0001) had higher mortality rates than in the control, and the mortality rate was higher in the hybrid treatment than in the *Bd*-GPL treatment (*p* = 0.0008). Mortality was first observed in frogs exposed to the hybrid on day 19 and 9/9 frogs (100%) died by day 34 (Fig. 1a). Mortality was first observed in frogs exposed to *Bd*-GPL on day 26 and 6/9 frogs (67%) died by the end of the experiment (Fig. 1a).

The ranked mortality by treatment for *I*. *parva* was: *Bd*-GPL, hybrid, and *Bd*-Brazil (*χ*^2^ = 36.723; d.f. = 3; *p* < 0.0001; Fig. 1b). Mortality in *I*. *parva* exposed to *Bd*-Brazil was similar to the control (*p* = 0.071; Fig. 1b). Mortality was first observed in frogs treated with *Bd*-Brazil on day 52 and 2/5 frogs (40%) died by the end of the experiment on day 58 (Fig. 1b). In contrast, frogs exposed to *Bd*-GPL (*p* < 0.0001) and the hybrid (*p* = 0.0003) had higher mortality rates than in the control, and the mortality rate was higher in the *Bd*-GPL treatment than in the hybrid treatment (*p* < 0.0001). Mortality was first observed in frogs exposed to *Bd*-GPL on day 18 and 6/6 frogs (100%) died by day 27 (Fig. 1b). Mortality was first observed in frogs exposed to the hybrid on day 31 and 6/7 frogs (86%) died by the end of the experiment (Fig. 1b). All direct-developing individuals that were not experimentally inoculated survived during both experiments.

**Figure 2:**
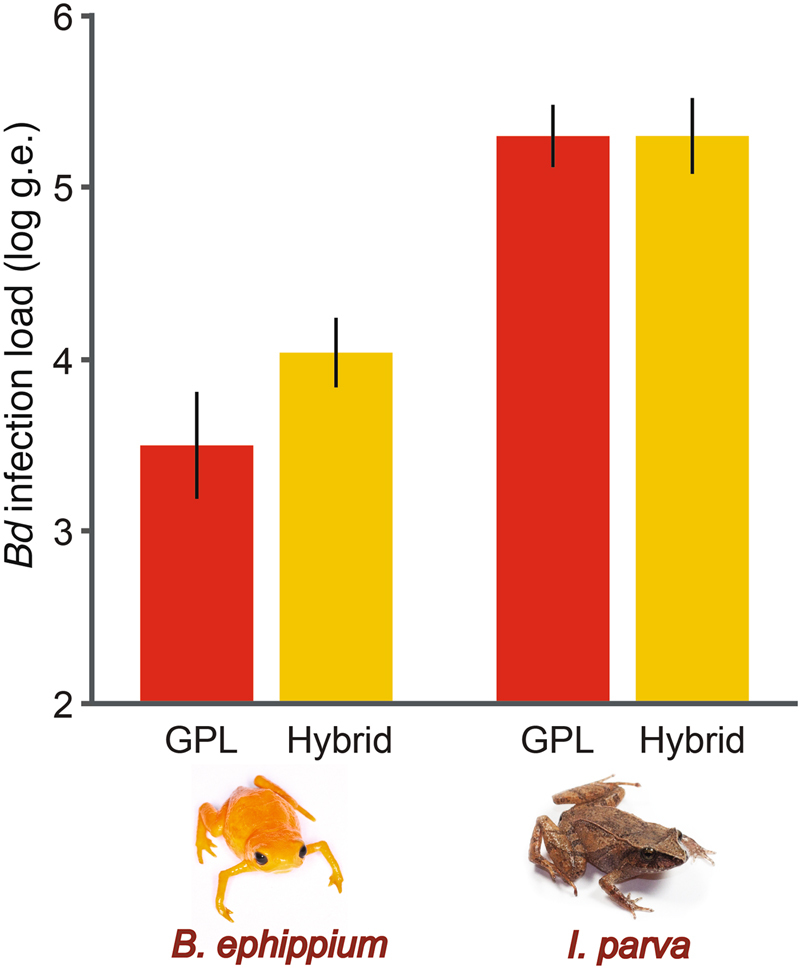
Average infection burdens (±SE) at the time of mortality for *Brachycephalus ephippium* and *Ischnocnema parva* inoculated with a hybrid genotype (yellow bars) and *Bd*-GPL (red bars).

Infection loads at the time of mortality were greater in frogs exposed to the hybrid than to *Bd*-GPL (*F*_[22,3]_ = 92.349; *r*^2^ = 0.926; *p* < 0.0001) but the magnitude of this effect was greater for *B*. *ephippium* than for *I*. *parva* (*t* = −2.89; *p* = 0.0084). For *B*. *ephippium*, frogs exposed to *Bd*-GPL had an average infection load (±SD) of 3,762 (±2,115) zoospore genome equivalents (g.e.) at the time of mortality (range = 1,116–6,555 g.e.), whereas frogs exposed to the hybrid had an average infection load of 12,441 (±6,091) g.e. at the time of mortality (range = 4,260–22,876 g.e.; Fig. 2). In contrast, infection loads at the time of mortality for *I*. *parva* were at least one order of magnitude higher than for *B*. *ephippium* and were more similar between *Bd*-GPL (mean ± SD = 213,961 ± 85,611 g.e.; range = 123,681–321,625 g.e.) and the hybrid (221,062 ± 109,100 g.e.; range = 2,303–409,665 g.e.; Fig. 2).

### Dendropsophus minutus *(host species withaquatic larvaldevelopment)*

We did not detect differences in mortality of *D*. *minutus* among infection treatments or between infection and control treatments (*χ*^2^ = 8.297; d.f. = 9, *p* = 0.504). Mortality was first observed in frogs exposed to *Bd*-Brazil on day 19 and 12/30 frogs (40%) died by the end of the experiment on day 60 (CLFT 041: 4/10 [40%], CLFT 142: 5/10 [50%], CLFT 150: 3/10 [30%]). Mortality was first observed in frogs exposed to *Bd*-GPL on day 10 and 14/30 frogs (47%) died by the end of the experiment (CLFT 073: 4/10 [40%], CLFT 131: 5/10 [50%], CLFT 137: 5/10 [50%]). Mortality was first observed in frogs inoculated with hybrid genotypes on day 14 and 13/30 frogs (43%) died by the end of the experiment (CLFT 024.2: 3/10 [30%], CLFT 160: 4/10 [40%], CLFT 039: 6/10 [60%]). Mortality was first observed in control frogs on day 22 and 3/20 frogs (15%) died by the end of the experiment.

**Figure 3:**
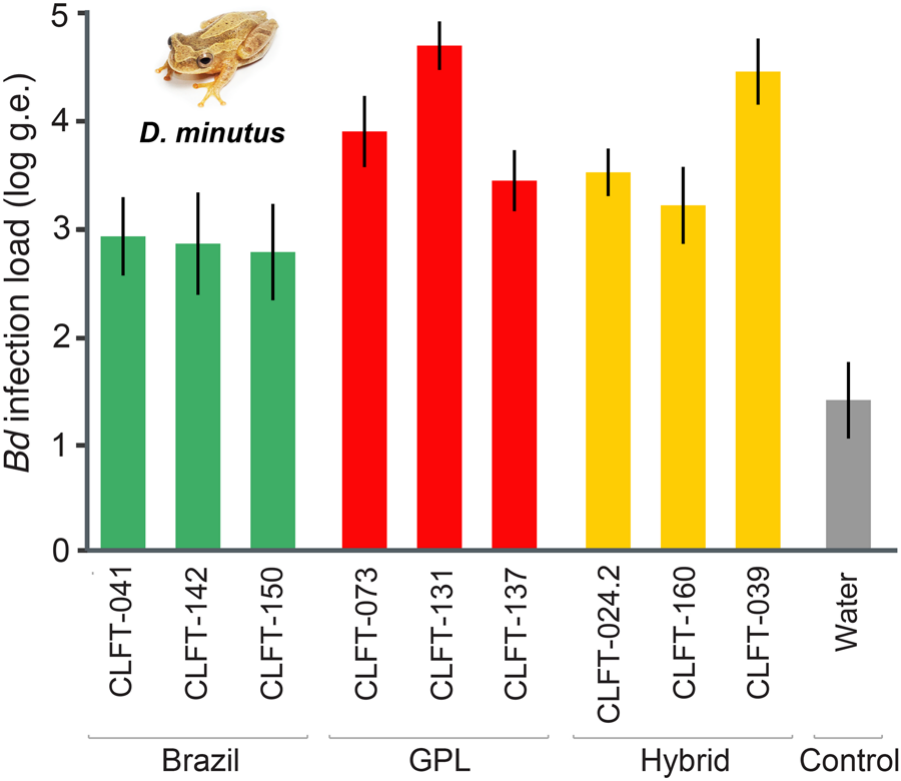
Average infection burdens 60 days post-inoculation (±SE) for *Dendropsophus minutus* inoculated with *Bd*-Brazil (green bars), *Bd*-GPL (red bars), and hybrid genotypes (yellow bars); each bar represents a different *Bd* genotype. Gray bar depicts natural infection burdens of the control group after 60 days incubation.

We detected differences in infection loads on day 60 within and among treatments (*p* < 0.001; Fig. 3). Average infection loads on frogs exposed to hybrids and *Bd*-GPL were highly variable within treatments (hybrids: 7,356 g.e.; 8,679 g.e.; 248,663 g.e.; *Bd*-GPL: 9,893 g.e.; 49,316 g.e.; 141,879 g.e.; Fig. 3). Average infection loads within the *Bd*-Brazil treatment were less variable and were generally lower than in frogs treated with hybrids and *Bd*-GPL (*Bd*-Brazil: 3,437 g.e.; 17,614 g.e.; 26,169 g.e.; Fig. 3). Thirteen of 20 control frogs carried natural *Bd* infections. Average infection loads on control frogs were lower than those on treatment frogs, likely reflecting what they were carrying in the field (Fig. 3). Passage rate was not a significant predictor of *Bd* infection loads, independent of *Bd* lineage (*F* = 0.228, *p* = 0.634).

## Discussion

Amphibian declines and extirpations throughout the Atlantic Forest have been attributed to *Bd*, and the zone of hybridization between *Bd*-Brazil and *Bd*-GPL is within the region with the clearest signature of disease-related mortality, underscoring the need to test the virulence of hybrid genotypes in native hosts^30,42^. In our direct-developing host species, mortality rates of frogs exposed to a hybrid genotype were higher (*B*. *ephippium*) or intermediate (*I*. *parva*) compared to *Bd*-Brazil and *Bd*-GPL. This finding indicates that hybrid virulence is context specific; in certain scenarios these genotypes may produce disease outcomes that far exceed parental ranges, while in others these genotypes may elicit intermediate disease outcomes. Since it is well-established that *Bd* has a dynamic genome and is now nearly globally distributed, this finding highlights the importance of global surveillance to detect future genomic shifts in this disease system that could lead to new outbreaks of chytridiomycosis, especially because a non-Brazilian host (*Lithobates sylvaticus*) also exhibited increased mortality in response to a *Bd* hybrid relative to the parental genotypes in a preliminary trial^51^. In addition, in other chytrid fungi, sexual reproduction produces a thick-walled resting stage that is capable of tolerating desiccation and high levels of heat and salinity^52^. Resting spores can also be produced by asexual reproduction, as in the chytrid *Rhizophydium brooksianum*^53^, which is in the same taxonomic order (Rhizophydiales) as *Bd*. No definitive evidence of a resting stage in *Bd* has been observed to date^54,55^, but this would have important implications for the geographic and temporal scales at which transmission of *Bd* could occur.

The relative virulence of hybrid genotypes and *Bd*-GPL appeared to depend on host factors, highlighting the context dependency of disease outcomes that is a hallmark of this disease system^56,57^. Compared to *I*. *parva*, *B*. *ephippium* died with relatively low *Bd* burdens, suggesting a defense tradeoff in which *B*. *ephippium* invested more resources in resistance defenses (minimizing parasite burden) at the expense of tolerance defenses (minimizing harm caused by parasites, such as mortality effects in this study)^58^. Theoretically, resistance strategies are expected to reduce pathogen fitness, eliciting a strong antagonistic host-pathogen co-evolution^59,60^. The effectiveness of an immune defense strategy that is heavily influenced by host-pathogen co-evolution should be positively correlated with the length of time that the host has been exposed to the parasite. In accordance with this expectation, *B*. *ephippium* exhibited the longest incubation periods and highest survival rate (variables indicating the most developed resistance defenses) when exposed to *Bd*-Brazil, the lineage with which this species has probably co-existed in the wild for the longest time period. It follows that incubation periods and survival were intermediate when *B*. *ephippium* was exposed to *Bd*-GPL, the lineage with which this species has probably co-existed in the wild for a time period intermediate between *Bd*-Brazil and hybrid genotypes. Lastly, this species exhibited the shortest incubation periods and lowest survival rate, as well as the highest infection burdens at the time of death (variables indicating the least developed resistance defenses), when exposed to hybrid genotypes, the group with which this species has probably co-existed in the wild for the shortest time period, or not at all.

These findings indicate that *B*. *ephippium* may be particularly vulnerable to infections by hybrid genotypes in nature, primarily due to the relative novelty of these genotypes. The vulnerability of *B*. *ephippium* to novel pathogens may be linked to its association with patchy, high-altitude habitats, which may have limited its exposure to pathogens throughout its evolutionary history or lowered its immunogenetic diversity^61–63^. In the case of *Bd*, anthropogenic habitat alteration may tip the host-pathogen balance further in favor of the pathogen, as deforestation may promote the evolutionary isolation of *B*. *ephippium* while at least some relatively novel genotypes of *Bd* appear highly competent at dispersing across even the most fragmented landscapes in the Atlantic Forest, such as our *B*. *ephippium* collection site at Jundiái^42^. Previous studies indicate that F1 hybrids often show hybrid vigor because of outbreeding enhancement, whereas F2 hybrids might express hybrid breakdown as a result of recombination^26^. Considering that all living *Bd* hybrid isolates are first-generation crosses (TYJ pers. comm.)^64^, we are unable to test whether our results for *B*. *ephippium* represent an instance of hybrid vigor that could weaken through subsequent crosses^16^. However, an F2 backcrossed to *Bd*-Brazil was observed in the field and its survival provides some indication that parasitemia of *Bd* hybrids may not be limited to the F1 generation^64^.

In contrast to *B*. *ephippium*, *I*. *parva* died with relatively high *Bd* burdens, suggesting the reverse defense tradeoff in which *I*. *parva* invested more resources in tolerance defenses at the expense of resistance defenses. Tolerance strategies are not expected to negatively affect the success of pathogen populations, so we would expect relatively weak co-evolutionary pressures between parasites and hosts that invest primarily in this type of defense strategy^59,60^. This may help to explain why the responses of *I*. *parva* to our panel of *Bd* treatments were not correlated with the relative length of time to which this species was probably exposed to each lineage in the wild. Rather, it is possible that this species was more responsive to the expression of hybrid phenotypes that were intermediate between the hypervirulent *Bd*-GPL and the hypovirulent *Bd*-Brazil, resulting in intermediate incubation periods and mortality in frogs exposed to the hybrid compared to *Bd*-GPL and *Bd*-Brazil. Our findings suggest that *I*. *parva* is particularly vulnerable to pathogens with aggressive phenotypes, such as *Bd*-GPL, regardless of the extent to which this species has shared an evolutionary history with co-occurring pathogens. Thus, an important avenue for future study is to determine the genetic or physiological factors that make *Bd*-GPL particularly damaging to hosts and the ecological backdrop against which these genotypes emerged. Immunological comparisons of disease progression among *Bd* genotypes and host species are necessary to verify the mechanisms we have proposed to explain the host-specific patterns in our data. Nevertheless, our results offer convincing evidence that both *Bd*-GPL and hybrid genotypes are virulent in the Atlantic Forest.

Compared to hybrid genotypes and *Bd*-GPL, frogs treated with *Bd*-Brazil had the lowest ranked mortality in both direct-developing host species and the lowest infection burdens in *D*. *minutus*, consistent with the hypothesis that *Bd*-Brazil has shared a long co-evolutionary history with endemic Brazilian frogs and is hypovirulent in endemic hosts^28,38,42,50^. A recent study also found that the *Bd*-Brazil genotype CLFT 001 (isolated from the Atlantic Forest) exhibited lower *in vitro* growth performance than the *Bd*-GPL genotype CJB5-2 and the *Bd*-Brazil genotype UM 142 (unknown geographic origin)^65^, another indication that the relative threat of endemic Atlantic Forest *Bd* genotypes is low. In contrast, the only other study that tested the relative virulence of *Bd*-Brazil and *Bd*-GPL reported that 50% of hosts died when exposed to *Bd*-Brazil and that the virulence of *Bd*-Brazil (one genotype) fell within the range of virulence shown by *Bd*-GPL (three genotypes)^66^. This conflicting study used the *Bd*-Brazil genotype UM 142, different *Bd*-GPL genotypes (from the eastern U.S. and Panama), and a North American host species. Our conflicting results may thus reflect host-independent variability in virulence within *Bd* lineages or suggest that *Bd*-Brazil has higher virulence toward non-native hosts. Evidence suggests that humans are facilitating the spread of *Bd* in South America^67^ and globally^38^. For instance, the *Bd*-Brazil genotype UM 142 was isolated from a bullfrog (*Lithobates catesbeianus*) collected from a U.S. amphibian market. We reiterate the concern raised by Becker *et al*.^66^ that lineages endemic to one region may lead to declines of naïve host populations in other regions.

Terrestrial, direct-developing frog species have typically been considered less vulnerable to chytridiomycosis than species with more aquatic life histories that may experience high levels of exposure to aquatic *Bd* zoospores^68–70^. However, both of our terrestrial study species acquired heavy *Bd* infections and experienced mortality from chytridiomycosis under laboratory conditions mimicking the microhabitat of direct-developing frogs in nature, consistent with laboratory data for other Brazilian direct-developing species^63^. In the wild, Brazilian direct-developers had high *Bd* infection loads^71,72^, but low infection prevalence^63^, the latter of which could be underestimated if frogs die quickly from infections or if sick frogs remain stationary in hidden refugia^63,73^. Even if low prevalence of *Bd* is currently facilitating population persistence of direct-developing host species in Brazil, this host-pathogen balance is precarious in an era of global change^61^. For example, in Puerto Rico, *Bd* dynamics in the direct-developing species *Eleutherodactylus coqui* shifted from enzootic to epizootic when extreme drought conditions associated with global climate change forced frogs to congregate in humid refugia, increasing transmission and reinfection rates^73,74^. Moreover, reduced levels of recruitment stemming from increased *Bd*-related mortality of juvenile *E*. *coqui* has led to recent, low-level population declines^75,76^, similar to other examples of negative population effects from *Bd* in the absence of drastic epizootic events^77–79^. Future research should investigate the potential effects of *Bd* on the population persistence of Brazil’s diverse direct-developing amphibian fauna, especially considering that the narrow geographic ranges of many Brazilian direct-developers leave them vulnerable to other natural and anthropogenic stressors and that *Bd* caused population declines of a direct-developing *Arthroleptis* in Cameroon^35^ and likely the extinction of several direct-developing species in the Atlantic Forest (see Supplementary Table S2).

In contrast to our direct-developing species, mortality rates of *D*. *minutus* were low across treatments, suggesting that this species is relatively tolerant to *Bd* regardless of variation in the genetic attributes of the fungus. This finding is consistent with previous studies of *D*. *minutus* in the laboratory^63,80^ and in the wild^81^. Patterns of infection loads in *D*. *minutus* by lineage matched mortality rates in our direct-developing species, with the lowest infection loads in frogs exposed to *Bd*-Brazil and higher infection loads in frogs exposed to *Bd*-GPL and hybrid genotypes, corroborating the evidence from our direct-developing species that *Bd*-Brazil is less virulent in native hosts. Infection loads were also less variable among genotypes within *Bd*-Brazil than within *Bd*-GPL and hybrid genotypes, which could reflect consistency in host immune responses stemming from long-term exposure to *Bd*-Brazil.

Our study shows that hybridization can be associated with high levels of virulence in *Bd*. Hybrid *Bd* genotypes emerged relatively recently in Brazil, at some point after the introduction of *Bd*-GPL to Brazil in the last few centuries^41^. The known distribution of hybrid genotypes is small (an isolated mountain range in the Atlantic Forest), which could suggest that it is functioning primarily as a parasite with intermediate rather than extreme fitness relative to parental populations and could be outcompeted by quick-dispersing *Bd*-GPL. However, it is unknown whether the current geographic distribution of hybrid genotypes is a product of their short evolutionary history (i.e., they might currently be spreading), their specific microhabitat requirements, or gaps in field sampling. Nevertheless, their coexistence with *Bd*-GPL suggests that they could be adept competitors in Brazilian landscapes. Applying newly developed, non-invasive techniques that can discriminate among *Bd* genotypes on skin swabs would help to paint a more complete picture of the spatial distribution of *Bd* lineages in Brazil as well as coinfection dynamics^82^. The same techniques could also be used with contemporary and retrospective sampling of preserved specimens to increase our understanding of which genotypes and lineages are associated with amphibian mortality in Brazil, track the spread of *Bd*-GPL through time, and determine whether the geographic distributions of hybrid genotypes and *Bd*-Brazil have changed over time^82^. Another useful avenue for future study is to determine how coexistence and hybridization of *Bd* genotypes in Brazil could influence host population recoveries through adaptive responses, which have been documented in other regions in the decades following mass declines^83,84^.

Our results do not point definitively to a single genetic culprit of *Bd*-related amphibian declines in Brazil. *Bd*-GPL is likely to have played a large role given its high level of virulence in some hosts and widespread geographic distribution^42^, but the hybrid zone overlaps the region with significant evidence of disease-related mortality^30^, and our results indicate that some host species are especially vulnerable to new genotypes, suggesting a possible role of hybrids in declines. Taking all these factors into account, a plausible scenario for disease-linked amphibian declines in Brazil is that the impacts of the introduction of *Bd*-GPL were exacerbated by the emergence of hybrids, possibly by overloading host immune systems with an even more genetically and spatiotemporally diverse assemblage of pathogen strains. As much as evolutionary novelty can aid species adaptation in an era of rapid environmental change, our findings underscore that this plasticity can also be advantageous for parasites, with serious consequences for the persistence of host populations^13,16^.

## Materials and Methods

### Study species

We selected three experimental host species with varying life histories and levels of tolerance (i.e., ability to minimize harm caused by parasites, such as mortality) to *Bd*. *Dendropsophus minutus* (Hylidae: Dendropsophinae) is a habitat generalist tree frog with indirect development and a close association with water bodies throughout its life history. The geographic range of this species covers most of tropical South America^85,86^, and it exhibits relatively high survival rates when challenged with *Bd*-GPL under laboratory conditions^63,80^. *Ischnocnema parva* and *Brachycephalus ephippium* (Brachycephalidae) are direct-developing leaf litter frogs that occur in southeastern Brazil^85^ and exhibit relatively low survival rates when challenged with *Bd*-GPL under laboratory conditions^63^. All three species are listed as Least Concern by the International Union for the Conservation of Nature^87^. In a vulnerability assessment of amphibians of the Brazilian Atlantic Forest, all three species were considered to have large geographic ranges and high local abundances but *D*. *minutus* and *I*. *parva* were classified as having wide habitat specificities, whereas *B*. *ephippium* was classified as having a narrow habitat specificity due to its association with high-altitude habitats^47^.

We collected adults of our study species in the municipality of Jundiaí, near Serra do Japi (*B*. *ephippium and D*. *minutus*) and in the municipality of São Luiz do Paraitinga, adjacent to Parque Estadual da Serra do Mar, Núcleo Santa Virgínia (*I*. *parva*), São Paulo state, Brazil. Both collection sites are located to the north of the *Bd* hybrid zone identified by Jenkinson *et al*.^41^. Some *D*. *minutus* tested positive for *Bd* at the time of collection but we elected not to treat infections to avoid any negative treatment-associated side effects and instead controlled for natural infections in our statistical analyses^88^. We only used *I*. *parva* and *B*. *ephippium* that tested negative for *Bd* in the field. For the experiments, frogs were randomly assigned to treatments and housed individually in plastic containers with sterile moist sphagnum (all species) and a sterile leaf for cover (*B*. *ephippium* and *I*. *parva*). Frogs were fed pinhead crickets *ad libitum* during the experiments.

### *Bd* isolates

*Bd* isolates were obtained from infected tadpoles sampled throughout the Atlantic Forest of Brazil from 2011 to 2015^42^. Tadpoles were screened in the field with a 10X hand lens for signs of chytrid infection by assessing the level of oral tissue dekeratinization^89^. Animals with signs of *Bd* infection were euthanized and oral tissues excised. Infected tissues were prepared for pathogen isolation on 1% tryptone agar with 0.2 mg.mL^−1^ penicillin-G and 0.4 mg.mL^−1^ streptomycin sulfate. Isolates of *Bd* were maintained on 1% tryptone agar at 21–23 °C until sufficient growth had occurred for DNA extraction. Isolates were genotyped to determine *Bd* lineage/group (*Bd*-Brazil, *Bd*-GPL, or hybrid) following the procedures described in Jenkinson *et al*.^41^. Cultures were maintained at 4 °C at Universidade Estadual de Campinas, UNICAMP, Brazil, and passaged every 4 mo.

### Challenge experiment

We cultured three hybrid, three *Bd*-Brazil, and four *Bd*-GPL genotypes (see Supplementary Table S1) in Petri dishes containing 1% tryptone agar at 19 °C for 7 d. The three hybrid genotypes represent all living hybrid isolates, and analyses of whole genome sequences indicate that all are F1 hybrids (TYJ pers. comm.)^64^. The *Bd*-Brazil and *Bd*-GPL genotypes were selected haphazardly. To inoculate frogs with *Bd* (day zero), we filled each Petri dish with 5 ml of distilled water for 30 minutes and scraped the substrate with a sterile scalpel to facilitate zoospore release. We then transferred the liquid contents of each dish to a sterile beaker, sampled 1 ml of the solution to quantify the zoospore concentration with a hemocytometer, and diluted the solution with distilled water to obtain the desired zoospore concentration for experimental inoculations.

We inoculated *D*. *minutus* with hybrid genotypes CLFT 024.2, CLFT 039, and CLFT 160; *Bd*-Brazil genotypes CLFT 041, CLFT 142, and CLFT 150; and *Bd*-GPL genotypes CLFT 073, CLFT 131, and CLFT 137. We inoculated 10 frogs with each genotype. Each frog was inoculated individually in a Petri dish containing 1.8 × 10^6^ zoospores in 2.5 ml of distilled water for 45 minutes. We exposed 20 additional individuals to the same volume of distilled water as *Bd*-unexposed controls.

We inoculated *B*. *ephippium* and *I*. *parva* with hybrid genotype CLFT 160 (*B*. *ephippium*: n = 9; *I*. *parva*: n = 7), *Bd*-Brazil genotype CLFT 150 (*B*. *ephippium*: n = 9; *I*. *parva*: n = 5), and *Bd*-GPL genotype CLFT 156 (*B*. *ephippium*: n = 9; *I*. *parva*: n = 6). Due to technical difficulties with preparing *Bd*-GPL for this component of the experiment, we were unable to use one of the three *Bd*-GPL genotypes that were tested with *D*. *minutus*. We inoculated each frog individually in a Petri dish containing 3.375 × 10^6^ zoospores in 1.5 ml of distilled water for 45 minutes. We exposed additional individuals to the same volume of distilled water as controls (*B*. *ephippium*: n = 8; *I*. *parva*: n = 6).

Survival was monitored daily, dead animals were noted, and dying animals were euthanized with an overdose of the anesthetic MS-222 if they showed lack of righting response, which is a typical sign of advanced stages of chytridiomycosis^90^. Dead animals were swabbed immediately following the protocol described by Hyatt *et al*.^91^. The experiment concluded on day 60 (*D*. *minutus*), 58 (*I*. *parva*), or 40 (*B*. *ephippium*; length of experiment truncated because this species can become stressed after long periods in captivity), at which point all remaining animals were swabbed and euthanized.

We extracted DNA from skin swabs using 50 ml PrepMan Ultra and screened samples for *Bd* presence and load using Taqman qPCR assays^92^. For *D*. *minutus*, we used *Bd* genotype-specific standard curves (for each genotype used in the experiment) ranging from 0.1 to 1000 zoospore genome equivalents (g.e.). For *B*. *ephippium* and *I*. *parva*, we built standard curves (0.1–1000 g.e.) using CLFT 159, a *Bd*-GPL genotype isolated from a *Hylodes* frog collected in the Atlantic Forest. We were unable to standardize infection load data for *B*. *ephippium* and *I*. *parva* due to unforeseen culturing difficulties with one of our genotypes, but we feel confident in using non-standardized infection loads in our analyses for these species because standardization did not influence the overall patterns in our data for *D*. *minutus* (see Supplementary Fig. S1).

All experimental protocols were approved by Instituto Chico Mendes de Conservação da Biodiversidade –Instituto Brasileiro do Meio Ambiente e dos Recursos Naturais Renováveis/Brazil (Permits 29964–11, 27745–13, and 57098–1) and the local Animal Care and Use Committee (Comissão de Ética no Uso de Animal – CEUA/UNESP permit #29/2016). Our experiment was carried out in accordance with all ethics guidelines and regulations.

### Statistical analyses

We used proportional hazards survival analyses^93^ to compare mortality rates among frogs exposed to *Bd*-Brazil, *Bd*-GPL, and hybrids, independently for each species. For *B*. *ephippium* and *I*. *parva*, we compared mortality rates among one genotype from each of the three treatments. For *D*. *minutus*, we compared mortality rates among nine *Bd* genotypes within the three treatments, including genotype as a fixed effect.

We used a Generalized Linear Mixed Model (GLMM) to compare infection loads on day 60 among *D*. *minutus* exposed to *Bd*-Brazil, *Bd*-GPL, and hybrids. In this model, we included the following explanatory variables: genotype, lineage/group, and the interaction between genotype and lineage/group as fixed effects, and infection load at the time of capture in the wild as a random effect. We also performed a GLMM to test for effects of passage rate on *Bd* loads of *D*. *minutus* with *Bd* lineage/group as a random effect.

For *B*. *ephippium* and *I*. *parva*, we performed independent General Linear Models (standard least squares) to compare infection loads at the time of mortality between frogs exposed to *Bd*-GPL and hybrids. For these models, we included log_10_-transformed infection loads as the response variable and *Bd* lineage/group (*Bd*-GPL or hybrid), host species (*B*. *ephippium* or *I*. *parva*), and the interaction between lineage/group and host species as explanatory variables.

### Data availability

The datasets generated and analyzed during the current study are available from the corresponding author on reasonable request.

## Electronic supplementary material

Supplementary Information
