# Supplementary material for: Hybrids of amphibian chytrid show high virulence in native hosts
Source: Sci Rep. 2018 Jun 25;8:9600. doi: 10.1038/s41598-018-27828-w (PMC6018099; doi:10.1038/s41598-018-27828-w)
Supplement: Supplementary file 1 — Supplementary Information [file 41598_2018_27828_MOESM1_ESM.docx]

**Hybrids of amphibian chytrid show high virulence in native hosts**

**Supplementary information**

S. E. Greenspan^1*^, C. Lambertini^2^, T. Carvalho^2^,

T. Y. James^3^, L. F. Toledo^2^, C. F. B. Haddad^4^, C. G. Becker^1*^

^1^Department of Biological Sciences, University of Alabama, Tuscaloosa, AL 35487, USA

^2^Laboratório de História Natural de Anfíbios Brasileiros (LaHNAB), Departamento de Biologia Animal, Universidade Estadual de Campinas, Campinas, SP 13083-862, Brazil

^3^Department of Ecology and Evolutionary Biology, University of Michigan, Ann Arbor, MI 48109 USA

^4^Department of Zoology and Aquaculture Center (CAUNESP); Universidade Estadual Paulista, Rio Claro, SP 13506-900, Brazil

**Supplementary Table S1.** *Batrachochytrium dendrobatidis* genotypes used in experimental inoculations. Locality (municipality, state), host species, year of isolation, and approximate number of passages are given for each genotype isolated from Atlantic Forest frogs.

| Genotype ID | Lineage | Locality | Host species | Year | Passages |
| --- | --- | --- | --- | --- | --- |
| CLFT 041 | *Bd-*Brazil | Morretes, PR | *Bokermannohyla hylax* | 2013 | 12 |
| CLFT 142 | *Bd-*Brazil | Morretes, PR | *Crossodactylus caramaschii* | 2014 | 8 |
| CLFT 150 | *Bd-*Brazil | Morretes, PR | *Hylodes cardosoi* | 2014 | 8 |
| CLFT 073 | *Bd-*GPL | Serra dos Órgãos, RJ | *Aplastodiscus* sp. | 2013 | 12 |
| CLFT 131 | *Bd-*GPL | Vargem Alta, ES | *Lithobates catesbeianus* | 2014 | 8 |
| CLFT 137 | *Bd-*GPL | Morretes, PR | *Hylodes cardosoi* | 2014 | 8 |
| CLFT 156 | *Bd-*GPL | Morretes, PR | *Hylodes cardosoi* | 2014 | 8 |
| CLFT 024.2 | Hybrid | Morretes, PR | *Hylodes cardosoi* | 2011 | 20 |
| CLFT 039 | Hybrid | Morretes, PR | *Bokermannohyla hylax* | 2013 | 12 |
| CLFT 160 | Hybrid | Morretes, PR | *Hylodes cardosoi* | 2015 | 5 |

**Supplementary Table S2.** Atlantic forest direct-developing anurans that declined or were locally extinct. Year indicates interval period or year of the last observed individuals.

| Species | Locality of the decline | Year | References |
| --- | --- | --- | --- |
| Brachycephalidae |  |  |  |
| *Brachycephalus alipioi* | Santa Teresa, ES | 1952 | Pombal & Gasparini 2006 |
| *Ischnocnema epipeda* | Santa Teresa, ES | 1978 | Heyer 1984 |
| *Ischnocnema oea* | Santa Teresa, ES | 1942 - 1984 | Carvalho et al. 2017; Heyer 1984 |
| *Ischnocnema paranaensis* | Antonina, PR | 1988 - 1999 | Carvalho et al. 2017; Bornschein et al. 2015; Eterovick et al. 2005 |
| *Ischnocnema parva* | Boraceia, SP | 1979 - 1983 | Carvalho et al. 2017; Heyer et al. 1988 |
| *Ischnocnema pusilla* | São José do Barreiro, SP | 1965 | Garey et al. 2014 |
| *Ischnocnema* sp. (aff. *guentheri*) | Boraceia, SP | 1979 - 1983 | Carvalho et al. 2017; Heyer et al. 1988 |
| Craugastoridae |  |  |  |
| *Holoaden bradei* | Itamonte, MG | 1978 | Carvalho et al. 2017; Stuart et al. 2008; DOU 2014 |
| Eleutherodactylidae |  |  |  |
| *Adelophryne baturitensis* | Pacoti, CE | 1993 - 1994 | Eterovick et al. 2005 |

**References for Supplementary Table S2**

Bornschein MR, Mauricio GN, Pie MR. 2015 Rectification of the type locality of *Ischnocnema paranaensis* (Anura: Brachycephalidae), a missing species of the Atlantic Forest of Brazil. Zootaxa, 3957, 249‒250.

Carvalho, T., Becker, C. G., & Toledo, L. F. 2017. Historical amphibian declines and extinctions in Brazil linked to chytridiomycosis. Proc. R. Soc. B 284 (1848): 20162254.

Diário Oficial da União. 2014 Lista Nacional Oficial das Espécies da Fauna Ameaçadas de Extinção. Seção 1, ISSN 1677‒7042, 121‒130.

Eterovick PC, de Queiroz Carnaval ACO, Borges‐Nojosa DM, Silvano DL, Segalla MV, Sazima I. 2005 Amphibian Declines in Brazil: An Overview1. Biotropica 37, 166c179.

Garey MV, Provete DB, Martins IA, Haddad CFB, Rossa-Feres DC. 2014. Anurans from the Serra da Bocaina National Park and surrounding buffer area, southeastern Brazil. Check Lists 10(2): 308‒316.

Heyer WR, Rand AS, da Cruz CAG, Peixoto OL. 1988 Decimations, extinctions, and colonizations of frog populations in southeast Brazil and their evolutionary implications. Biotropica 20, 230‒235.

Heyer WR. 1984 Variation, systematics, and zoogeography of *Eleutherodactylus guentheri* and closely related species (Amphibia: Anura: Leptodactylidae). Smithsonian Institution Press. 402, 32‒33.

Pombal Jr. JP, Gasparini JL. 2006. A new *Brachycephalus* (Anura: Brachycephalidae) from the Atlantic rainforest of Espírito Santo, southeastern Brazil. South American Journal of Herpetology, 1(2): 87‒93.

Stuart SN, Hoffmann M, Chanson J, Cox N, Berridge R, Ramani P, Young B.  2008 Threatened Amphibians of the World. Barcelona, Spain; International Union for the Conservation of Nature, Gland. Switzerland; Conservation International, Arlington, Virginia, USA.: Lynx Editions.

**Supplementary Figure S1.** Average Infection burdens (± SE) for *Dendropsophus minutus* inoculated with *Bd*-Brazil (green bars), *Bd*- *Bd*-GPL (red bars), Hybrid (yellow bars), and water (grey bar) calculated using a single *Bd* genotypic standard (CLFT 159).

**
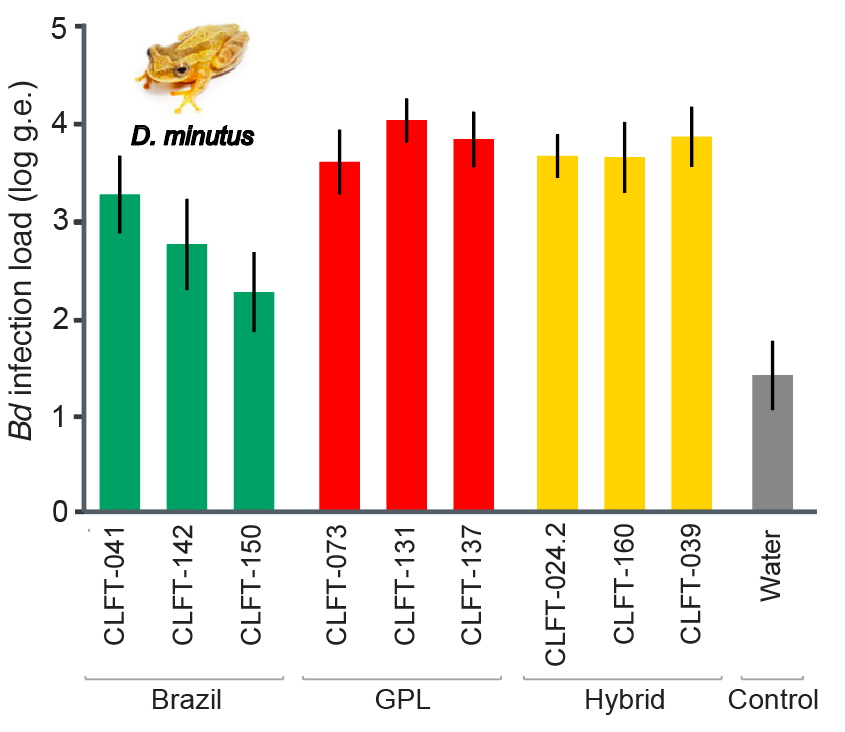
**
